# The history and welfare of working mules in the valleys of the Toubkal massif, in the High Atlas of Morocco

**DOI:** 10.3389/fvets.2023.1256501

**Published:** 2023-10-13

**Authors:** Glen Cousquer, Hassan Alyakine, Victoria Lindsay-McGee

**Affiliations:** ^1^Royal (Dick) School of Veterinary Studies, University of Edinburgh, Scotland, United Kingdom; ^2^School of GeoSciences, University of Edinburgh, Edinburgh, United Kingdom; ^3^Institut Agronomique et Vétérinaire Hassan II, Madinat Al Irfane, Rabat, Morocco

**Keywords:** pack mules, working equine welfare, mountain tourism, mountain development, High Atlas, One Health, wounds, bitting injuries

## Abstract

The appearance of the mule in the remote villages of the Toubkal National Park in the High Atlas Mountains of Morocco can be traced back to the early years of the twentieth century. The mule population’s subsequent growth in numbers accompanied the shift away from a subsistence economy that was made possible by the appearance and growth of the mining and mountain tourism industries. This paper reviews this early history, drawing on the accounts provided by early explorers and anthropologists, before developing a mixed methods approach to evaluating the welfare of pack mules in two villages within the National Park. The first village is part of the main access route to the Toubkal, the principle summit of the National Park and therefore much visited. The second is a much more remote, under visited and less developed village. Ethnographic work, studying muleteering practice and undertaken over several years is reported here and supplemented by findings from a detailed survey of the mules of both villages. This allows the work undertaken and the lived realities of the working mule across several generations of inhabitants to be presented together with data about the primary welfare concerns identified on clinical examination. In village one, 72 mule owners and their mules were surveyed and examined. Many of these mules worked in tourism, providing a source of revenue for their families. This work was often undertaken by teenagers/young adults working their father’s mules. Tethering was widely practised and evidence of tethering injuries were identified in most mules. In the more remote village, 18 owners and their equids were surveyed and examined. In this population, mules were more likely to be worked locally in agriculture, building and to collect firewood. Bitting injuries associated with the use of the traditional bit were a significant concern in both villages. The universal use of a closed shoe was also in evidence in both villages; this was associated with atrophy of the frog and hoof imbalances. The reasoning for the use of the traditional bit and closed shoe are presented and alternatives discussed.

## Introduction

The emergence of the mule’s role as a beast of burden working in mountain tourism is founded on our appreciation of this species’ great attributes (1, 2) as a means of transport in remote mountain environments, where road and other transport infrastructures have failed to penetrate (3–6). These attributes prompted Alphonse Guénon (1) to propose that, if the camel is the ship of the desert, the mule is the ship of the mountains (p. 29). Today, the mountain tourism industry employs mules across the world, from the Alps to the Andes, the Himalayas to the High Atlas (5–9). Our appreciation of mules does not always extend to their care and welfare, however, for whilst we are proficient at exploiting their services, we are much less proficient at repaying them for their work in any reciprocal sense. Where lack of resources and insufficient knowledge coincides with a harsh working environment, welfare often suffers and this inattention to working animal welfare is particularly true of the mountain tourism industry in Morocco (6, 10–14), where this study is situated.

**Figure 1:**
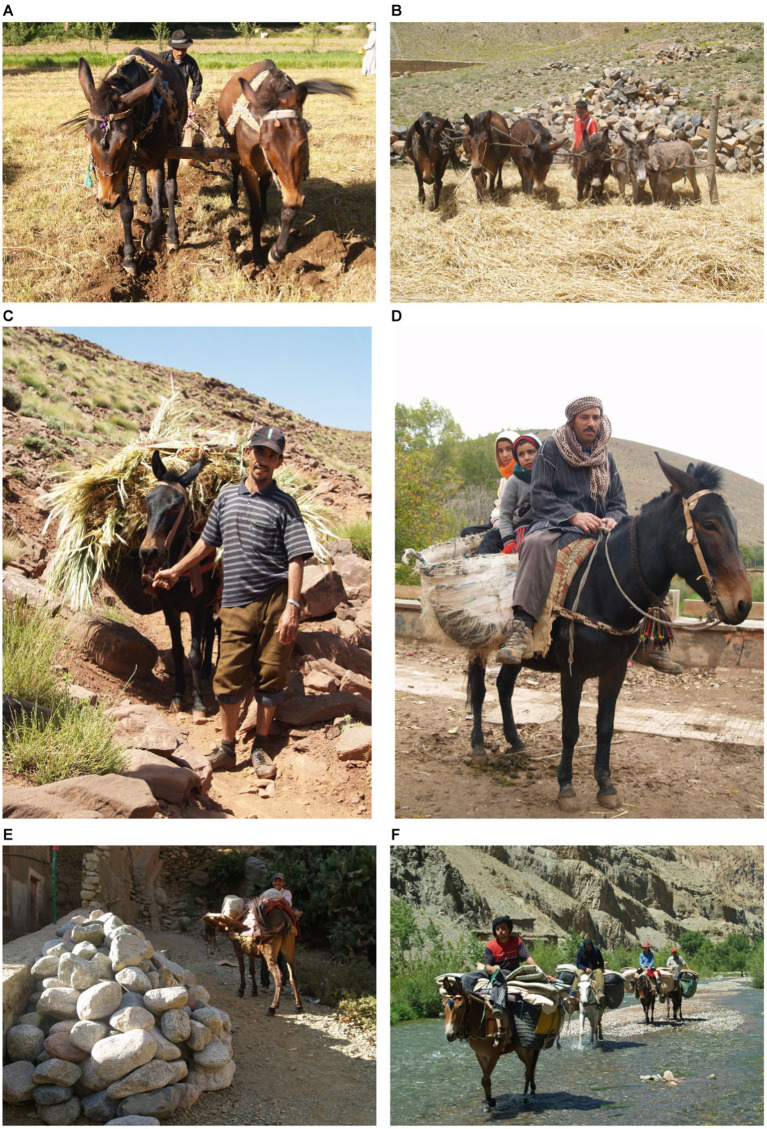
Traditional life in the High Atlas sees the mule working in agriculture: ploughing **(A)**, threshing **(B)**, transporting fodder **(C)** and people **(D)**. Three images are from the Aït Bouguemmez valley, the fourth **(C)** was taken above the village of Magdaz, in the Tessaout valley and illustrates the daily need for fodder to be cut and brought back to feed the animals housed within the village. **(E)** Working in construction: in the Ourika valley, motorised transport and roads are present and can be used to transport building materials. Even here the mule is still required to deliver materials directly to the building site, which may be some distance from the track. These materials may now consist of bricks and blocks, rather than the more traditional earth and stone, but the mule’s versatility and utility ensure that the local populations remain as reliant as ever on this ‘work horse’. **(F)** Mules in mountain tourism: the three muleteers pictured in the Tessaout valley, in 2008, have chosen to ride their loaded mules along the riverbed and have offered a ride to a tired client on the spare mule. Here spare does not mean unladen but rather unridden, for the mules are carrying all the trekking and camping equipment for a party of six trekkers, their guide and three muleteers.

Today, in many areas of the High Atlas, mules continue to be employed to plough the fields, thresh the corn, carry fodder and, on market day, ensure the transport of his master and all goods to and from the souk (Figures 1A–D). The mule also carries building supplies (Figure 1E), gas bottles and other less traditional household items, such as beds, sofas and even fridges! During the trekking season (Figure 1F), the mule will also find employment carrying the luggage of trekkers and other visiting tourists. Mules therefore continue to play their own part in establishing themselves as essential workers and travel companions, demonstrating unrivalled work capacities and the resilience to endure great hardship. These unusual attributes have long been recognised, with Williams and Speelman (15) writing, in 1948, that:

Those who are staunch supporters of the mule say that, in comparison with the horse he will live longer, endure more work and hardship, require less attention and feed, is less liable to digestive disorders, lameness and disease, is more easily handled in large numbers, is less irritable, and is more capable of performing work in the hands of a mediocre or poor horseman. Whether or not all these claims may be substantiated, it is a fact that the mule is well established as a work animal in those sections where climatic conditions are severe, suitable feed often lacking and horsemanship not a prevailing art (p. 2)

The remote valleys of the High Atlas of Morocco are certainly characterised by a severe climate (16), lack of grazing (17–19) and an absence of skilled horsemanship (10). The Toubkal National Park was established in 1942 under the French Protectorate (20, 21) and since then has become a very popular tourist destination (22–24), in part because of its proximity to Marrakech but also because of the appeal of climbing North Africa’s highest mountain, the Djebel Toubkal. This has led to an increase in the mule population in the Mizane Valley, which leads to Imlil, principle trailhead for those wanting to climb this mountain. The Toubkal rises to a height of 4,167 m and its valleys fall steeply and are prone to flash flooding, which means that soils are virtually non-existent on the highly eroded slopes (25). Eroded sediments accumulate in the narrow valleys, aided by the creation of walled terraces to retain a depth of soil on which crops can be grown (25, 26).

The valley’s human population has also grown and their economy has diversified, with fruit orchards and tourism now playing a significant role in household economics (26). The mule population are caught up in all these changes and their welfare is a product of a complex interplay of historical, socio-cultural, socio-economic, educational, geographical and other factors. This is further compounded by the challenges posed by climate change (27). Any detailed understanding of the inter-relationships between such factors and the resulting health and welfare issues seen in the local mules calls for a comprehensive and in-depth study. This paper sets out to deliver an in-depth account of some of the various threads that make up the current tapestry of life of the working mules in this part of the High Atlas.

**Figure 2:**
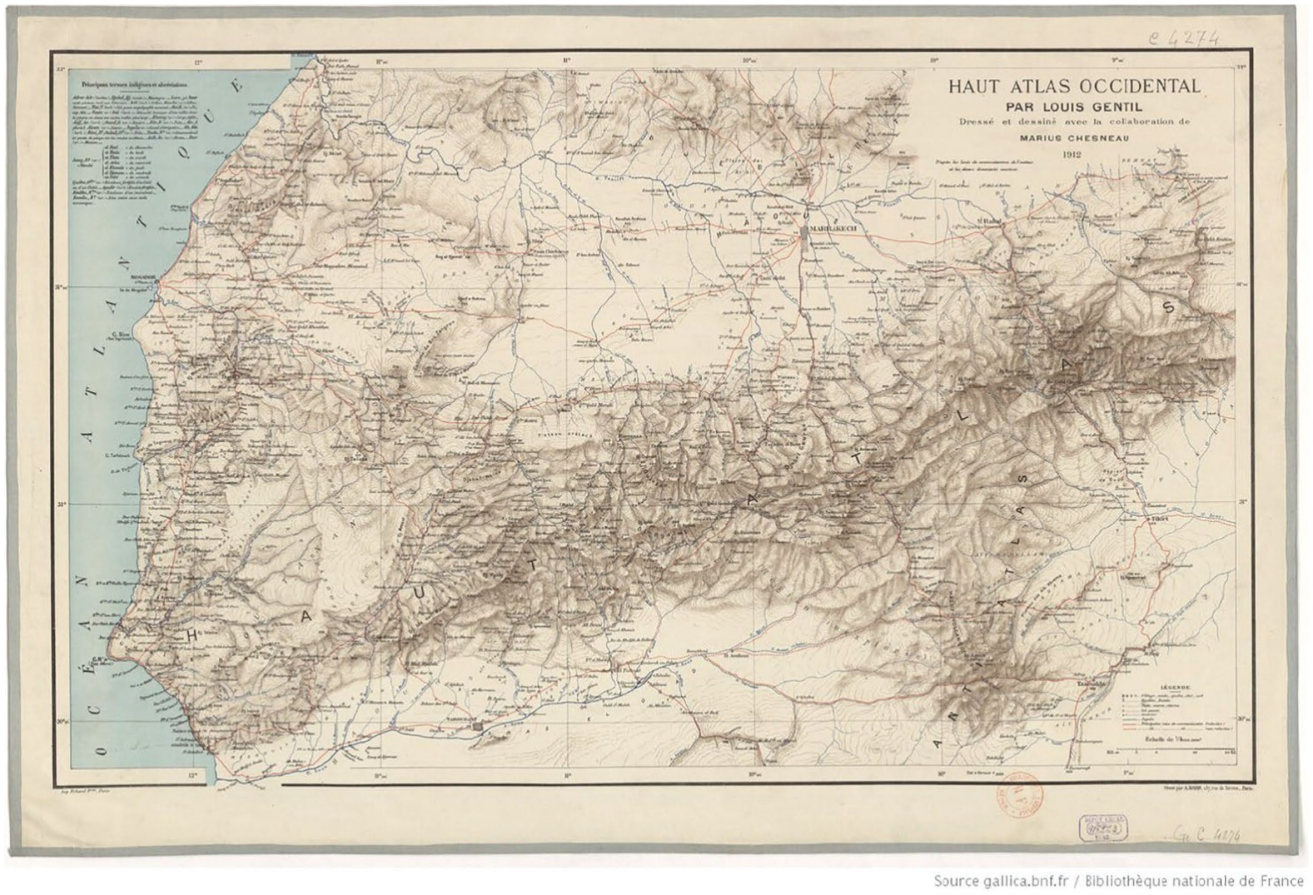
The Western High Atlas of Morocco stretches in a curve to the south of Marrakech. The red line running north to south in the centre of the map links Marrakech to the Toubkal massif. Source: Gallica.bnf.fr (Bibliothèque Nationale de France).

In seeking to develop a richer sense of the complex entanglement(s) (28–31) of factors contributing to the ways health and welfare materialise for the mule, we have chosen first to present an exploration of the mule’s appearance in these valleys and the working lives they have had. This is informed by insights from ethnographic work conducted within the community between 2013–2017 and an accompanying exploration of the historical ethnographic literature. The paper is thus primarily ethnographic in nature but is supplemented by findings from survey work conducted, in 2014, in two different villages (Douars). The first Douar (village) is that of Aremd, one of four villages clustered around the trailhead at Imlil, within the Mizane valley (Figure 2). The second Douar is called Tizi Oussem and is located in a neighbouring valley, that of the Azzaden. This valley is much less visited and has been less impacted by mountain tourism; this allows potential differences in husbandry, working practices, health and welfare to be explored.

This mixed methods approach allows us to consider the following research questions:

- How has muleteering emerged and evolved in the Mizane and Azzaden valleys?
- What does life for a working mule in the Mizane and Azzaden valley consist of?
- What husbandry practices are seen in these two valleys?
- What health and welfare concerns are seen in these two valleys and what are the likely underlying causes?

The materials and methods that allowed these strands of data to be collected and analysed in order to explore these questions are presented next. There then follows an empirical section in which key findings are presented and discussed together, as is common practice in ethnographic work. The paper closes with an extended discussion in which we try to make sense of these elements and what they may offer in the way of insights that can help develop priorities for working equid health and welfare interventions.

## Materials and methods

In this section, we provide an outline of the research approach taken in this study. This not only explicates the three data collection parts of this project and how they inform each other; it also emphasises the methodology underpinning this work. In this study the welfare of working mules is conceived ontologically as an enacted networking that arises in the moment as a result of the geo-socio-culturally situated practises of those, including the mule, who enact welfare (31). This network is a system that cannot be reduced to its constituent parts and analysed in a reductive, lytic sense for we then lose sight of the whole. It must be understood as an emergent system of relational elements that, together, construct the whole and are also available for deconstruction.

The three elements of fieldwork whilst presented below in succession, were in many respects concurrent as findings arising in one area prompted exploration of another area. An ethnographic retracing of a visit to an abandoned mine with a local who had worked there with his mule thus prompted this area of literature to be searched, whilst the discovery of an old account of the traverse of a remote col. prompted this journey’s re-creation with a local guide and muleteer in order to experience and study the elements that such approaches make available (31). The surveying work involved daily walks up to Aremd and several day-long treks across a high col. to reach Tizi Oussem. These three approaches reflect different ways of knowing that each contribute to our understanding of the health and welfare concerns of working pack mules in the Mizane and Azzaden valleys. The constructivist methodology must be appreciated in order to see how the following methods each help us develop this richer understanding and appreciation of the contexts within which any relatively objective findings are situated.

### Ethnographic walking and reading

It is widely acknowledged that any body of literature should be approached from a historical perspective when conducting a literature review (32). This does not, however, simply equate to providing a disciplined chronological account for there is a need to situate the literature within its historical contexts and to critically examine the history of the topic itself (33). Georgiou (32) proposes a series of eight questions that can help in reading into a historical body of literature (pp. 267–8) and can “facilitate a progressive appraisal of the integration of history within a literature review, one that can feed back into itself and thus allow for corrections, amplifications, new questions, and even new discoveries” (p. 267). Whilst it is emphasised that these need not be followed exactly and can be changed substantially, we have held them in mind when seeking to explore the emergence of muleteering in these valleys. In particular, we have tried not to judge previous research but rather to understand the objectives, whilst remaining sensitive to changes in nomenclature and remaining sensitive to the relations between older and more recent literature. We thus recognise that the earliest records of visits to these remote valleys arose within a disparate set of practices that predated the work of the socio-anthropologists who subsequently studied the area (34). These practices include early exploration, military expeditions, mining, and alpinism to name a few. Hassan Rachik (34) reminds us to consider the positionality, theoretical ideas and biases of the researcher and that these influence what is considered worth observing, questioning and reporting on as well as how it is reported.

For the purposes of this paper, the exploration of the literature involved not just reading into the peer reviewed literature but visiting the archives of the Club Alpin Français de Casablanca (31, p. 107) and seeking out early accounts of expeditions^1^ (in French and English) and reading them carefully in order to identify mentions (typically fleeting) of mules and other working equines and any insights they might offer on the role of mules in local communities, the care they received and their welfare. Developments that impacted on the use of mules, including mining, road building and the development of alpinism and then mountain tourism were also explored. This meant the literature review was unsystematic and exploratory, open to surprising mentions appearing in unlikely places and new insights arising as the literature was related to the ethnographic experiences on the ground. It is these we consider next:

Any ethnographic study of mule welfare in the mountain tourism industry involves journeying through time and space. Such ethnographies are, by definition, multi-sited (35), focussing both on the animal and the emergent human-animal relations and understandings that arise through and in such forms of itinerant living and their accompanying practices. These journeys unfold and are thus amenable to study. Cousquer [31, pp. 102–108] provides a detailed account of how field sites were accessed and insights gained into the history of muleteering that could then be related back to the monographs, guidebooks, articles and papers consulted. The field notes and thesis produced as a result of this have been reviewed retrospectively with a view to answering the research questions considered in this paper.

### Survey

The opportunity to carry out a systematic survey of the mule population and mule owners arose when we were asked to supervise a DMV^2^ research project. In developing this idea, we chose to focus on the largest of the four villages in Imlil that has been exposed to the phenomenon of the Toubkal Trail and a village in the Azzaden valley. One of the elders of the village of Aremd who has since become the President of the local muleteer association, coordinated the study ensuring all members of both villages who owned a mule participated. The work was conducted over 2 months (March and April 2014), during which time two treks over were undertaken to Tizzi Oussem for multi-day stays in the village.

The survey consisted of an interview and, in the majority of cases, a home visit and a basic clinical examination. The survey work was undertaken by a single individual who was fluent in French and Arabic and therefore able to speak to the villagers without the need of a translator. The President of the local association was available to help with checking particular terms in Berber, where such clarifications were needed. Supervision of the individual conducting the field work was provided to improve reliability but it was not possible, in the time frame, to assess inter-observer reliability. Interviews were not recorded; instead findings were recorded on paper and subsequently uploaded to a database. The interview of the owner or a member of the family sought to develop a picture of the mule’s life including:

- When the mule was purchased/length of ownership?
- Where the mule was purchased and at what age?
- Who worked the mule?
- What work was undertaken and in what proportions?
- Foot care including date of last shoeing, frequency of shoeing, and foot care.
- Dietary information – food fed and amount fed.
- Watering practices.
- Where stabled, size of stable and stable management?
- Rest periods and what this consisted of including whether allowed free time?
- Tethering practices.
- Contact with other mules and livestock.

The clinical examination sought to establish/confirm:

- The mule’s age (<5 years/5–15 years/>15 years).
- Sex (male or female).
- Coat colour (bay, grey, or chestnut).
- Weight, following the protocol developed by Kay et al. (36).^3^
- Attitude when approached, a hand placed under chin and walked.
- Body condition score (0–5) (37).
- Conformation of the midline (back and withers).
- Presence of wounds.
- Presence of dental hooks.
- Foot examination.
- Lameness.

Answers were captured in a notebook and subsequently uploaded into an Access database. Data was cleaned, categorised and analysed quantitatively in Python 3.7. Plots were produced using the seaborn package (38) and Chi square tests of association between variables carried out using the scipy package (39) *chi2_contingency* command.

### Ethics statement

The survey part of this study was planned and undertaken at a time when the IAV had yet to establish their *Comité Ethique* and so the study could not be submitted for ethical review. The analysis of the data collected that we have undertaken for this paper is, however, a prospective study using an existing data set. This analysis was therefore submitted to the University of Edinburgh’s Veterinary Ethical Review Committee (VERC), within the Royal (Dick) School of Veterinary Science, for approval in 2023 (Reference: 107.23). The ethnographic part of this study is reported retrospectively here. The original study was approved by the Ethical Committee of the University of Edinburgh’s School of Geosciences.

## Results

Relevant elements of the ethnographic study are presented first, followed by key results from the survey work. The former will, by its very nature be discursive and will help to situate and contextualise the survey results. These too are discussed as this is consistent with the narrative sense-making developed here.

### Retracing the early history of muleteering on the Toubkal

The emergence and evolution of muleteering in the High Atlas of Morocco can be discerned in the writings of early explorers. In Morocco, the mountainous interior fascinated many such explorers, including Buffa (40), Cuninghame-Graham (41), Harris (42), Rohlfs (43), and de Foucauld (44).^4^ Travel and tourism are, however, dependent on safety, with early travellers being provided with armed escorts or guides to ensure safe passage (41, 45, 46). According to Boujrouf et al. (47, p. 69), tourists only arrived in the massif after its pacification. The High Atlas has long been a mountain fortress, serving as sanctuary and refuge to those who lived there (48, p. 141) and resisting the incursions of both alpinists and occupying forces. Unsurprisingly, it was one of the last parts of Morocco to be mapped, with some areas remaining blank until the 1930s (49, 50). As recently as 1917, 5 years after the creation of the French protectorate, only those areas in which topographers could venture as part of a military column had been surveyed, leading the topographer Théophylle-Jean Delaye to describe these as completely unknown and closed to Europeans (49, pp. 3–4).^5^

According to Boujrouf et al. (47, p. 73), the Atlas was, during the 1920–30s appropriated by alpinists-*cum*-scientists. Their various efforts paved the way to the creation, in 1942, of Morocco’s first National Park, the Toubkal National Park (24, 54) and the emergence of mountain tourism from the 1920s onwards (55, pp. 224–225). The history of mountaineering and subsequently mountain tourism in the High Atlas can be traced back to the pioneering activities of a small group of alpinists and the founding, in 1922, of the Moroccan High Atlas section of the French Alpine Club (CAF). This led, the following year, to the first ascent of the Djebel Toubkal (55). Somewhere in all these explorations mules started to make an appearance.

**Figure 3:**
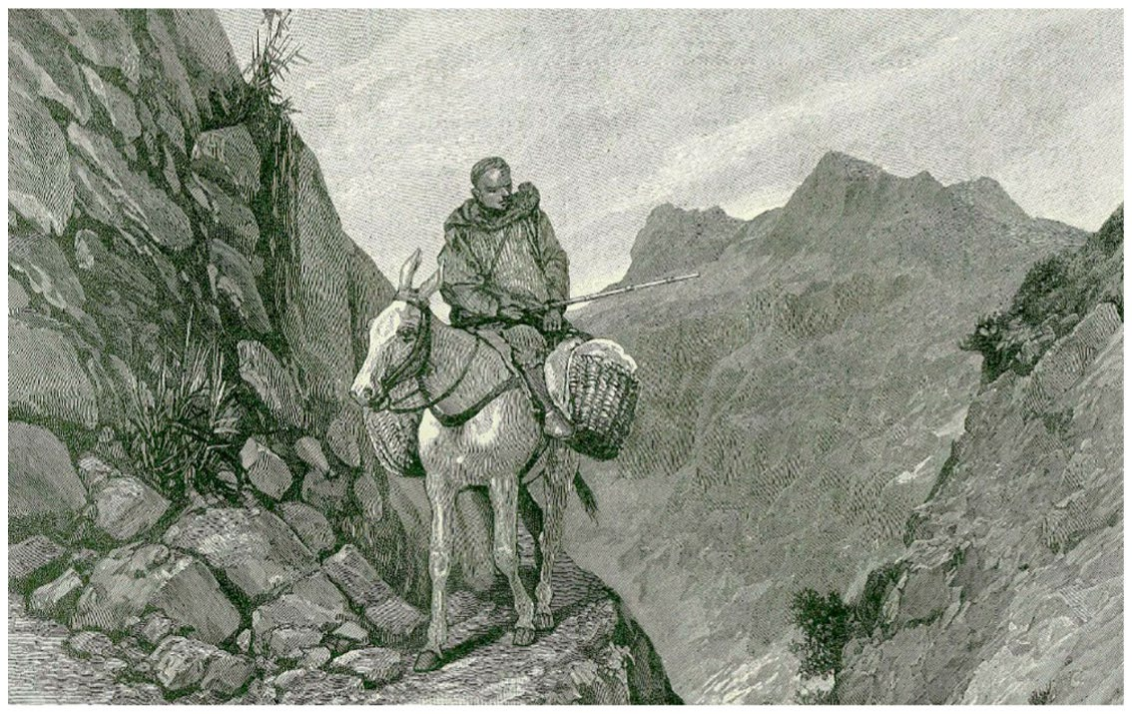
The mountain path by Caton Woodville (56) illustrates the narrowness of mule paths and the steep and exposed terrain mules work on.

**Figure 4:**
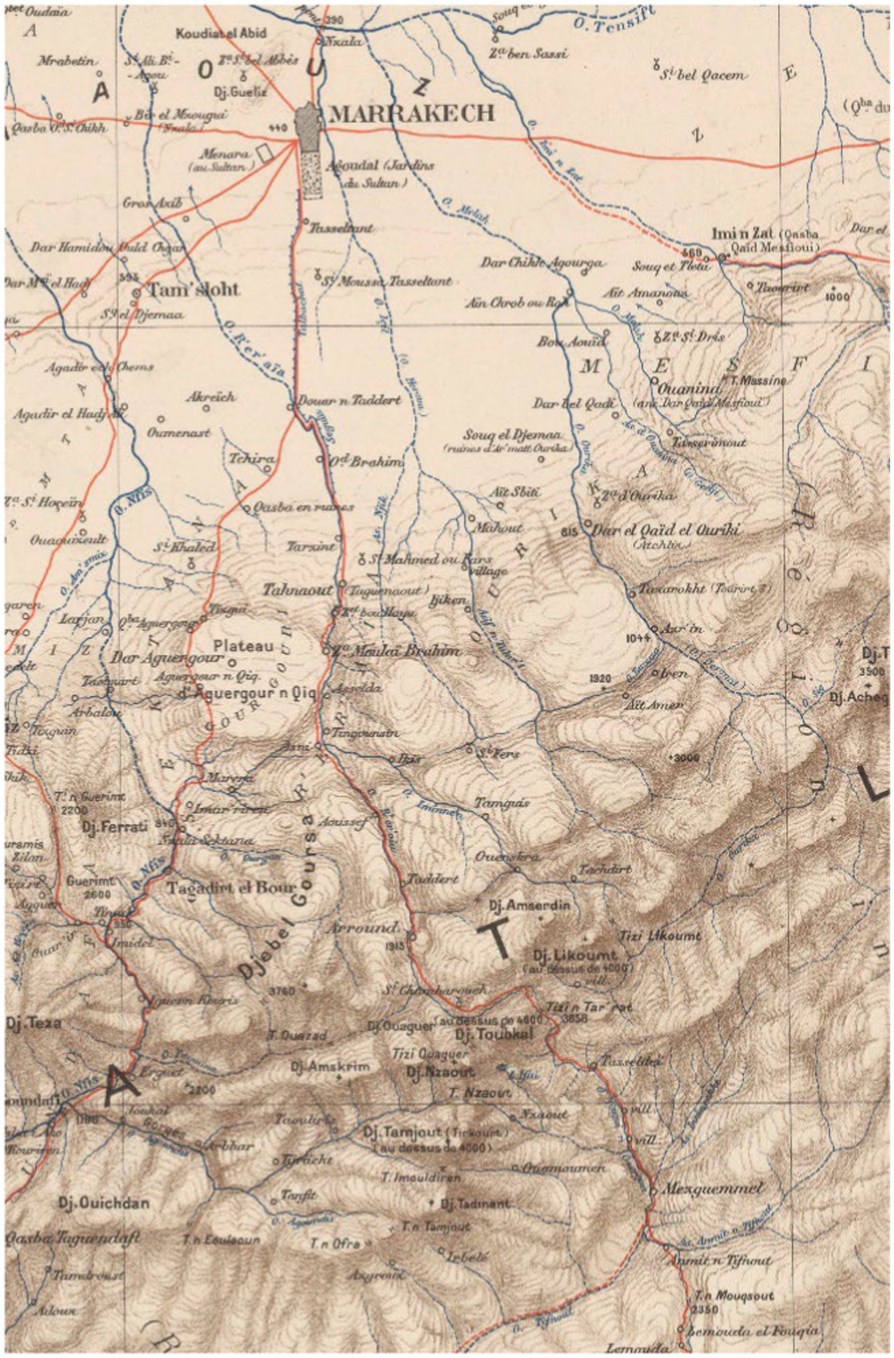
A close up of Louis Gentil’s (45) map of the western High Atlas shows a red line descending from Marrakech, passing though Asni and Aremd (spelled here Arround) before turning east at Sidi Chamharouch to cross the Tizi n Tarharat (spelled here Tizi n Tar’ret). The village of Tizi Oussem and the valley of the Azzaden are not indicated on this map. The Source: Gallica.bnf.fr (Bibliothèque Nationale de France).

An 1892 print, by Caton Woodville, entitled “The Mountain Path” is one of Woodville’s scenes without occidental tourists from his time in Morocco (56, p.93). It depicts the narrow mule paths that mules and their riders are likely to have had to contend with (Figure 3). One of the earliest accounts of travel into the challenging terrain that lies above Aremd was written in 1919 by Paul Penet (45) and tells of his traverse of the Tizi n Tarharat (3,460 m) in 1917.^6^ This is one of a number of high cols in the Toubkal area and is shown as the main route from Marrakech south across the High Atlas, on the 1932 map by Louis Gentil (Figures 2, 4), passing through Asni and Aremd before crossing over the Tarharat to reach the remote village of Tissaldi in the Tifnout valley. Penet’s account of his group’s voyage is insightful for it tells us much about the terrain, how local villagers travelled across it and what was transported. He writes (45, p. 8):^7^

We climbed little by little; everyone dismounted except Si Abd en Nebi, the envoy of Si el Madani, who remained on his horse. He was all pale, altitude sickness and palpitations made walking impossible for him.

At 3,100 m we stopped, man and beast needing a rest. It was cold. For the first time our lungs drew in the delicious cold air that fell towards us from the ridges. A spring offered us ice-cold water (6^°^). At the edge of the path we saw several dry stone shelters where travellers would seek shelter from snow storms or for the night. They also serve to shelter livestock as the people of the Tifnit do not hesitate to drive their cattle across the Tizi – Tar’ret to sell them in Marrakech.

Two men on foot joined our group: bare headed, in rags, one with goat hair slippers on his feet, the other sandals made from walnut wood. They were returning to the upper TIfnout after 6 days away. They had been to Moulay–Brahim (a 3 days walk) to buy two measures of maize that they were carrying on their backs. These people are incredible.

His account of this journey emphasises how unsuitable this route (and the terrain more generally) is for horses, that the local villagers travel everywhere on foot and that mules and horses are a luxury (45, p. 13):

The horse of Si Abd en Nebi lay in a neighbouring encolosure. Laminitic and colicing, he was in a bad way. The owner appeared disinterested and we found ourselves insisting the horse be rubbed, covered and treated.

… After a day as hard as yesterday’s, our animals had earnt a rest. Our schedule fortunately meant that today’s stage was very short.

Aguezrane where we were due to camp that night was no more than 8 km as the crow flies. But the path we took was so abominable, hugging at times the granite mountainside then snaking in the bed of the torrent when it was not in the torrent itself, that our journey took three hours. To say that this path serves the villages that are strung the length of this valley is an exaggerated euphemism; and it is precisely around the villages that the paths are most difficult. We realised that the habit of these mountain people is to travel on foot. Owning a mule, let alone a horse is an insolent luxury that only the chiefs can afford. Our caravan of nine equines, ten with that of the chief who came out to meet us, is so unusual that the terraces were thronged with curious faces as we passed.

The Tarharat is one of a number of cols close to or above 3,500 m surrounding the Toubkal, together with the Likemt (3,555 m), the Ouanoums (3,664 m), and the Ouagane (3,750 m). As the lowest, it was the route of choice for villagers from the Tifnout heading to the souks. Penet (45, p. 21) emphasises that the difficulty of the paths is such that the principle exports from these valleys are walnuts and livestock and these are sold in Marrakech and Taroudant. Wool is not exported for it is consumed locally. Over the next few decades, these isolated villages were increasingly influenced by the outside world, prompting Dresch and Lepiney (55, p. 38) to write that:

The villages lost in the depths of their valleys no longer live in isolation, in a self-sufficient subsistence economy. The markets and the distant big city are a permanent temptation. And prices change. Why? The mountain inhabitant knows little of the national or international economies. The reality is that he has to spend more, no matter how much he argues over each and every penny; more sometimes than his meagre budget allows for. And so he must sell more and more.

The common thread in these different historical strands is that they are all related to a fundamental imperative – that of selling to raise money… But what can these mountain communities sell? Historically, they have sold walnuts, almonds, livestock, walnut roots and a range of other saleable commodities. Iris bulbs became valuable once they became sought after by the perfume industry. And those that had nothing to sell would try to sell their knowledge or their labour or, if they were lucky enough to have a mule, that of their mule. Fundamentally, these communities have to find things to sell. And then they have to find buyers…

**Figure 5:**
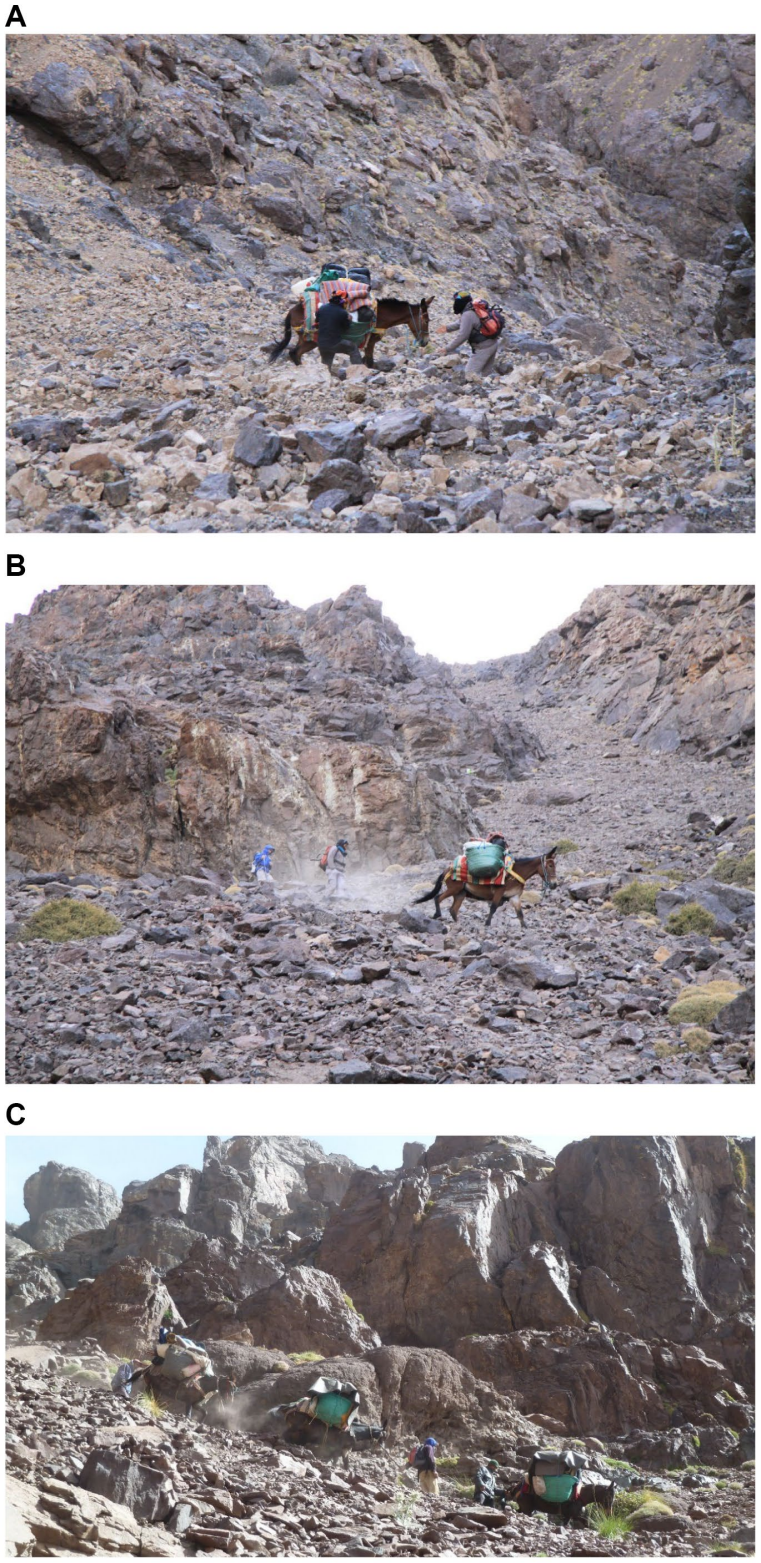
**(A)** The guide and muleteer intervene to rebalance the mule’s load as she descends the upper section of the scree at the top of the Tizi n Tarharat. **(B)** Looking back up the Tizi n Tarharat: the nature of this steep ground and rocky scree can have a heavy impact on the mule – giving rise to pack saddle sores, tendon strains, wounds and foot injuries. **(C)** A train of mules and muleteers working on a trek descend towards the Lac d’Ifni in the Tifnout valley, having crossed the Tizi Ouanoums. This ground is steep and unforgiving and the loads are heavy. All these mules are worked in traditional bits. Note how the mule handler in the centre of the image has created a noose around the neck of his mule to pull her forward, whereas other handlers are working their mules from behind.

During my field work, I crisscrossed these high cols, paying close attention to the terrain, to the challenges it posed, to how local muleteers worked their mules over this ground and to what was being transported and why (Figures 5A–C).

The nature of the saleable goods carried has extended from walnuts to include almonds, iris bulbs, walnut tree roots, and fruit including apples and cherries and other produce grown in these high valleys. Fodder and firewood can be carried by mules but it is often the womenfolk who will undertake this work. The main reason for mules to appear in these high valleys can be traced, therefore, to the appearance of first mining and then mountain tourism. Penet (45, p. 15) tells of a local Jew who shows them samples from a secret local mine that he is willing to sell to them. According to Moret (57, p. 262) Morocco had a reputation for being a fabulous mining Eldorado. This was largely based on the simple testimony of indigenous people who were thus transmitting the memories of the Roman and Portuguese miners, of old.

The development of mining operations in and around the Toubkal massif was more than just folkloric. There was living memory and experience of it on the ground. Mohamed was born in 1950 in Imlil, the village just below that of Aremd. He told of how he went to work in the mines at the age of fifteen because his father was unable to work. He did not want to work in the mine at Tadart, situated high up above the Neltner refuge (3,207 m) on the flanks of Aguelzim (3,680 m). This mine was reputed to be very tough as there was no mule path up to the mine. Consequently, the miners would carry the 50 kg sacks of mineral down to the Neltner refuge where they were loaded onto mules. He said that the miners might earn seven dirhams a day (approximately £0.53).^8^ The mules would have had to carry their load down to Imlil, which only became accessible by piste when this was completed in 1956 (58, p. 24).^9^

Another local informant who had worked in the copper mines above Aremd took me to see where he worked and explain what was involved. Omar’s story is typical of someone who grew up in the valley. As we walked the old and vanishing mule paths together, he told me how he had spent much of his early working life on the steep and unforgiving mountainside above the village. We were heading up to visit the abandoned copper mines above Tidli.^10^ He had worked there as a muleteer for 3 years in succession (1991–1993), leaving at 5 am to arrive at the mine for 7 am. He would often give one of the five miners that accompanied him a ride on his mule. He would then load two sacks of mineral ore, each weighing between 75 and 100 kg, onto his mule’s back and head back to Imlil, where he would arrive at about 11 am. There he would unload and receive a chit for his work. At the end of the month, when the lorry arrived from Marrakech to pick up the mineral, he would be paid, receiving a meagre 17 dirhams per day.^11^

I asked him about the load on the mule and whether she suffered any injuries. He was of the opinion that, in those days, the mules were much stronger – partly because they were better fed. There was much more barley being produced in the valley in those days and this suffered with the switch to fruit trees.^12^

The arrival of apple and cherry trees and the high cost of buying in barley has, he explained, meant that “some owners underfeed their mules and some are even reduced to giving them spaghetti!”^13^ ^14^

The paths we explored together had all but disappeared, reclaimed by the hillside as one rock slide after another had conspired with broom bushes to render the evidence of the mules’ passage invisible. Omar, however, could retrace the path for it was etched in his memory, as it had been in his mule’s.^15^ Thus, as we followed what was once his daily commute, a picture emerged of the precarious existence these men had carved out for themselves.

I learnt that Omar’s father had worked a lot in this mine and that they had worked together. This was at the end of the mine’s working life. The miners had used explosives and drills to work the mine and, impressively, there had been no accidents. The only accident occurred after the mine had closed when a local guide fell into one of the shafts and suffered injuries from which he was to die.^16^

I asked Omar why they chose this work and heard him say

… in those days there was no choice. There were very few fruit trees and little or no work in tourism. When the mine closed in 1994, … he went back to shepherding for a few years before … finding work in tourism as a muleteer and then, eventually, as a cook.^17^

At the mines, Omar explained to me that the mules

… once loaded, would not come back along the path we had just tried to follow. This, he said, was too steep. Instead they climbed up to the col above us (the Tizi Oufzdad) and from there dropped down to a much better path that contoured round the mountain.^18^

We followed this route, climbing over the col. before dropping to the *azibs* (shepherd huts and sheep folds):

We descended to the azib – a veritable scree run over difficult ground, all cut up by water channels. He explained to me that he had spent years of his life tending sheep and goats up here and that the azib belonged to his family.

Today, nobody in Achayn wants to shepherd. He said it wasn’t clean (‘propre’) and the life was too hard. They now employ a shepherd from Tizi Oussem to come over and look after the sheep; his family now only keeps one sheep at home.

Thus, as one source of income closes, another opens. Today, Omar has managed to secure safe, regular employment as a chef, at a local hotel. This means he has a guaranteed income and is in receipt of social security.^19^ He is also able to supplement this income by renting out his two spare rooms to visiting Moroccan tourists, earning him 150 MAD per room, per night. Life is thus considerably better than it once was and he will be able to send his children to school.

Omar’s story is echoed by others of his generation. In Aremd, there are only a few old shepherds left, no one is continuing the tradition; the shepherds stepping into their shoes come from neighbouring valleys – and in this case, specifically the second village in this study, Tizi Oussem. As Fabrice Cuzin explains when talking about generational change in the villages: “The inheritance of a way of life is broken; … the old no longer serve as a reference for the next generation. There are no apprenticeships”.^20^ Michèle Salmona (59, pp. 93–97) emphasises the importance to animal welfare of life-long apprenticeships in peasant societies: Whether speaking of the Peul or the French, the transmission of animal knowledge from one generation to the next necessitates the selection of those with an aptitude for this work and the development of a slow, gentle, patient disposition and fortitude in the face of hardships and isolation. This transmission and, in particular, the affective component, is lost when children go away to school or attend college later in life to learn about the technical (*I-It*) aspects of animal production (59, p. 97). The young of the valley are all striving to establish new lives and forge new paths for themselves and their families. But how does one escape from poverty? What mules and mule welfares does this absencing enact?

Where historically artisanal mining had offered opportunities to earn hard currency (57, 60, 61), today other opportunities have emerged. Those who complete their baccalaureate can go on to university and can apply to the guide school (57) with a view to training and qualifying as a mountain guide. The ambitious aspire to set up agencies and partner with a foreign agency who will send them clients. Many set up gîtes, adding to the supply of accommodation that has seen Imlil offer nearly as many B&B bed nights (47) as Casablanca (57) on TripAdvisor!^21^ This uncontrolled construction programme (62) has, arguably, blighted the peaceful character of the valley in what Goodwin (63, pp. 18–22) has described as a “tragedy of the commons.” Those with language skills but no qualifications can find work as receptionists, waiters, drivers and faux guides. Options are limited, however, for the young, unmarried men, who have not completed their schooling. During harvest time, employment can be found bringing in the walnuts, cherries and apples.^22^ This, however, does not provide a regular income stream. The lucky ones find work locally in shops, hotels, restaurants and cafés; many though find themselves leaving to find work in the cities. Some travel down to Dakhla to find work as paid agricultural labourers, others seek work in Casablanca and other cities or further afield.

De Sinety captures the paradox of the rural exodus that sees young Berbers of the Atlas descending to the cities where they find themselves shipwrecked in an alien world where capitalism and individualism hold sway, a world that the visiting tourist is anxious to flee:

They had to leave their valleys in the High and Anti Atlas that were no longer able to feed them. Washed up in noisy, aggressive and polluted cities, far from the solidarity of their village communities, they are forced to confront the apprenticeship of an urban capitalist society with its emphasis on individualism.

A minimum material need for subsistence leads these young Chleuhs to the city, whilst a spiritual quest leads privileged westerners to leave them. Our paths cross, our motivations are opposed, but we can still meet. (64, pp. 7–8)

Spaak (65, p. 228) similarly comments on the socio-cultural insularism that renders it difficult for the people of the High Atlas to leave their villages behind and venture down onto the foreign, anonymous plains where life is easy and a man’s bond no longer has the same value. Necessity; however; forces them to leave to find work (66). The exodus of over 50% of menfolk from these communities is; according to Spaak (65, p. 230), as grave a concern as desertification. He highlights the developments in agriculture that could help stem this “haemorrhaging,” whilst also referring to the promise offered by exploiting other resources including minerals, walnut wood and tourism. Since then, the High Atlas has been transformed by the development of opportunities to earn hard currency from fruit farming and mountain tourism^23^ (26, 61) and from the attempts to turn local resources into heritage objects (68, 69).

This has not, however, been without its problems for the environment (24, 61) and for communities, which once cohesive and well-ordered are increasingly divided by competition (68, 69). Tourism has thus allowed those who became guides and gîte owners to make their fortune. But what of those left behind? The unlucky ones – those from poor families, with little or no education – are typically reduced to working as muleteers. Those who have escaped this fate, recognise how lucky they are. A faux guide and driver whose brother still works as a muleteer acknowledges the extent to which muleteers are exploited by the industry:

The muleteers do a lot … I don’t mind if they only pay us 200 dirhams and the other 50 goes back to the muleteer … because they do most of the things … what am I doing? It’s nothing I take my bag and go walking, I have nuts, water, oranges, I can stop wherever I want and chat with the guests. But the muleteers have to charge the mules … walk so quick to pass you and you have to find them already making your lunch and then they have to charge the mules and keep going again to reach the final destination. Once he gets there he has to feed his mule, wash, make the dinner. They are doing lots of things for nothing. The payment for the muleteer is nothing at all. Those people deserve more than that. They deserve double price; they deserve even 180 or 200 dirhams.^24^

The muleteer’s unenviable status places them just above shepherds: at the bottom of the pile. Some spend hours waiting for clients, earning as little as 10 MAD to carry suitcases from the village up to one of the hotels or gîtes. The mules wait too; standing tethered, with their pack saddles on, denied food and water, unable to escape the heat and the tormenting flies.

During the summer, the influx of Moroccan tourists seeking relief from the heat of Marrakech (71, p. 6) provide a stream of potential clients and opportunities to earn 100 MAD for doing the trip to Sidi Chamharouch, potentially 200 MAD if two trips can be squeezed into a day.^25^ These ‘touristes internes’ often negotiate hard and show little concern for mule welfare. On a trip, up to Sidi Chamharouch, Hamid, a muleteer from Aremd, said:

… foreigners were much readier to think about the mule (they liked animals more). He explained this by saying that they would save their scraps for the mule. By contrast, Moroccan tourists would often say that his mule was no good and ask him to hit it in order to make it go faster … He refused to do so and said: “Je ne l’ai pas trouvé dans une boite de Vache Qui Rit”.^26^

Muleteers seeking such work also must contend with the influx of men and boys from other valleys, desperate to secure work and willing to undercut the typical daily rate.

The muleteers has [sic] to be all together. If they are not together they are not making any deal. … If Imlil decide to go for 150 dirhams, but Tachedirt, Oukaïmeden, Ourika they can still come to work for the same price. They have to send a letter to each village in the Atlas who has muleteers who work with tourists … if anyone move without this price … then from there, there can be …. It will be difficult but if they work with each other and behave each other they can get to that point.^27^

During the summer months, many muleteers purchase an old worn out mule and flog her hard through the summer to scrape a living. This can mean denying her rest during that time, then selling her on to avoid paying to feed her through the winter.

On the track to Aït Aïssa, and again on the climb up to the Tizi n’Oudite, Hamed told me about the challenges of finding work in Imlil. According to him, in his village of Aran, there are only two people who have proper jobs … They are employees … they have a certain income. The other men of the village have to find work where they can. For him, this means working hard with a mule through the summer and putting some money by to get him through the winter. He has worked in the cities (both Marrakech and Casablanca) – in pizza restaurants. In Imlil, the men have little to do, however, other than the seasonal work that agriculture and tourism throws their way.^28^

Hajj, a village elder, explains the problems this can give rise to

Tu connais la région, tu connais les gens. Tu vas trouver il y a quelqu’un qui est pauvre … acheter un mulet pour 3000 dirhams. … Un mauvais mulet … Mais qu’est-ce qu’il fait parce qu’il a besoin pour le travail … pour gagner pour la famille. Lui, il pense seulement gagner un peu d’argent. Il part sans penser pour la mule. Tu connais les mules qui sont mort dans la montagne c’est les mules pour les pauvres ‘man’ qui a acheté la mule pour 3,000 dirhams, 4,000 dirhams.^29^

This is life on the margins. The precarious existence led by those struggling to eke out a living is, largely invisible to the visitors and agencies; the consequences on the mule are therefore viewed not through the lens of phoric hardship described by Salmona (59) but through that of the disapproving tourist and employer. Such has been the challenge of understanding the welfare of equines in the Arab world, since Daumas (72), in the mid-1800s, cautioned against judging too quickly:

… beaucoup des personnes ont conclu que ce peuple n’avait aucune connaissance des vrais principes hippiques; elles lui ont même refusé tout amour du cheval. C’est qu’elles n’ont point voulu réfléchir que, tantôt pour sauver leurs familles, tantôt pour conserver leurs biens, et souvent pour obéir aux lois de la guerre sainte (djéhad), ces mêmes Arabes … étaient forcés de se servir de leurs chevaux en raison des besoins qu’ils éprouvaient, des circonstances qui les dominaient; mais ils savaient parfaitement qu’il eût été préférable de ne point agir ainsi.^30^ (72, pp. 151–152)

These then are the geo-historical, ecological, socio-economic, and cultural contexts within which any exploration and evaluation of working mule welfare in the valleys around the Toubkal are situated. We now turn to and consider a selection of findings from the villages of Aremd and Tizi Oussem to further deepen our understanding or working equine welfare.

### The working life of mules and muleteers in Aremd and Tizi Oussem

**Figure 6:**
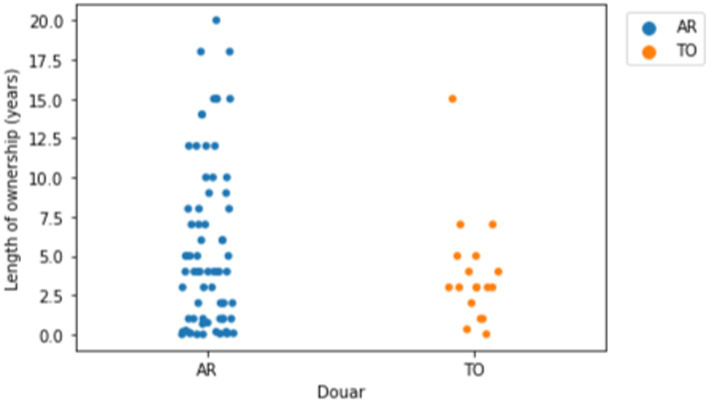
Strip plot of length of ownership in years by Douar (village). Mean length of ownership in AR was 5.53 years (*n* = 72) and in TO was 3.85 years (*n* = 18).

The human population in Aremd (AR) is approximately 1,900–2,000 people, whilst in Tizi Oussem (TO) it is estimated to be 600–670.^31^ The survey documented at least 72 mules living in Aremd. All mules were female, except for one gelding. By contrast, in Tizi Oussem (TO), a much smaller village, sixteen mules were documented, all female; as well as two male donkeys (Table 1). Owners attributed this choice to the fact female mules are calmer, more patient and easier to work with. Mules in both villages were predominantly aged 5–15 years with only five and three mules over the age of fifteen reported in each village. In Aremd, seventeen owners had owned their mule more than 10 years and three for close to 20 years (Figure 6). Mean length of ownership was 5.5 years in AR and 3.9 years in TO. This reflects the typical working age of mules working intensively on this terrain – historically in the mining industry and today in the mountain tourism industry. Mules who are no longer able to earn their keep are sold to work in the cities. A dignified retirement is not an option and owners recoup the monetary value of a mule with little thought as to how their mule might end her days working in the cities. This is a serious welfare concern for these aged mules move from carrying loads to pulling carts on busy city streets.

**Table 1:** Differing demographic data of mules between mules in Aremd and Tizi Oussem.

		AR	TO
Sex	Female	71	16
	Male	1	2 (donkeys)
Age	<5 years	9	2
	5–15 years	56	13
	>15 years	5	3
Bought out with the Douar	Yes	6	8
	No	66	10
Mule handler	Owner	57	11
	Owner and family	2	5
	Owner’s family	10	2
	Owner and others	2	0
Mules working in construction	Yes	3	3
	No	28	0
Mules engaged in tourism as their only work	Yes	12	0
	No	18	3

**Figure 7:**
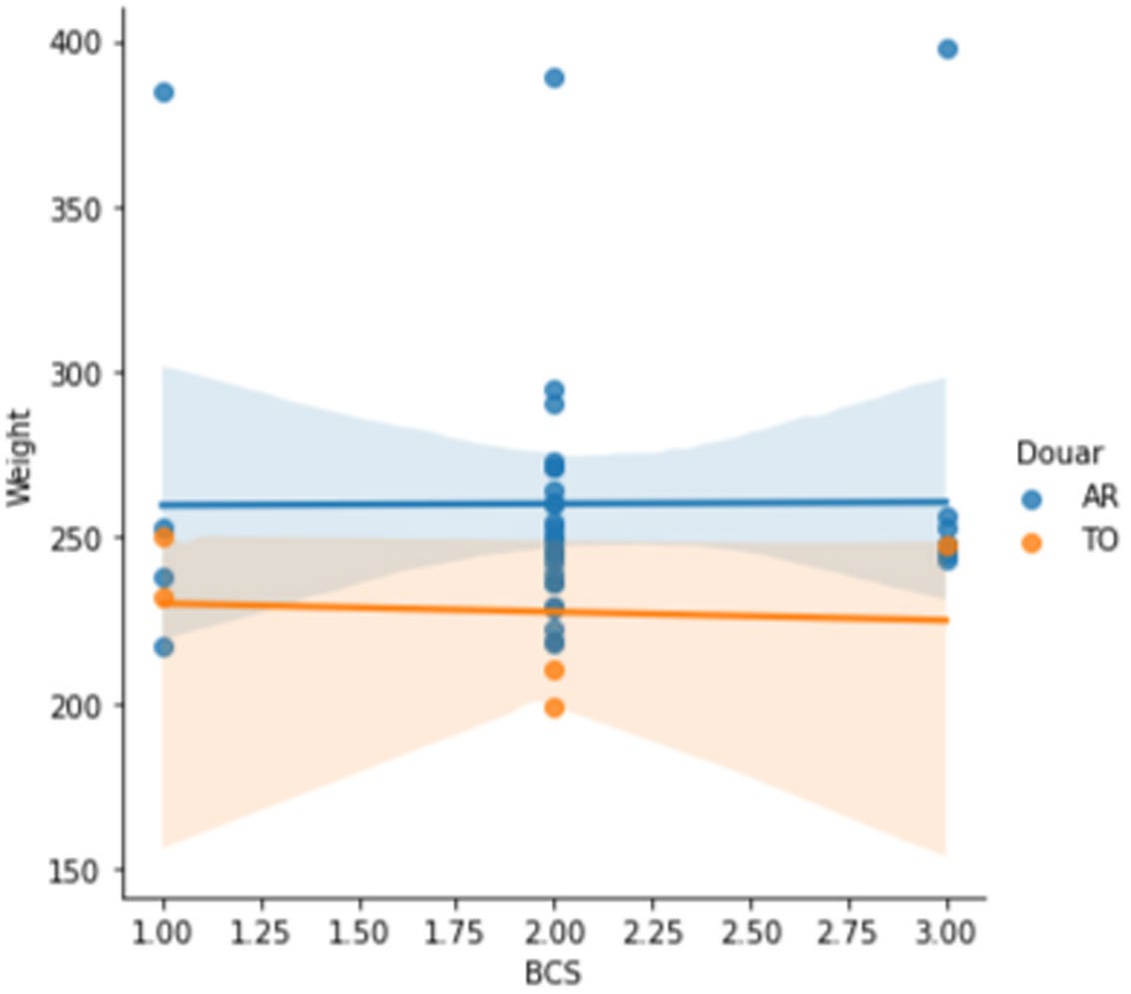
Regression plot illustrating relationship between BCS and weight of mules with a linear regression line per Douar (village). Mean BCS (on a 0 to 5 scale) in AR was 2.1 (*n* = 72) and TO was 1.8 (*n* = 18). The regression lines showed no close relationship between BCS and weight.

Mules in TO were significantly more likely (*χ*^2^ = 14.30, *p* = 0.0064) to be bought from within the village than mules in AR, where 91.7% of mules were bought from outwith the Douar (Figure 7A), with the market in Asni the most common purchase place (38 mules). Mule owners had also travelled to more distant souks, including those of Marrakech (3), Amssouzart (4), Setti Fatma (3), Agoundiss (3), Imintanout (3), and Tifnout (5) to purchase their mules. In TO, 55.6% of mules were bought from outwith the Douar, the most common purchase place was TO itself (8 mules), however. This reflects the fact that there are no horses in either valley and no mule breeding therefore occurs in the area.

Bay mules appeared to be more common or popular than grey mules in Aremd, where a single chestnut mule was identified. The weight of mules ranged between a minimum of 192 and a maximum of 397 kg (mean in AR was 259.8 kg s.d. 41.4 kg, in TO was 211.1 kg s.d. 44.4 kg). The significance of the low weight and size of the mules may reflect a preference locally for smaller mules when working in steep terrain. This data is also helpful as a guide for establishing weight limits where responsible tour operators develop mule welfare charters (13). Body condition score was generally low in both villages (Figure 7) which may reflect the lack of grazing, access to food and workload as well as other causal factors including poor dentition. Poor dentition, including the appearance of dental hooks, is likely to arise from the lack of grazing opportunities and the emphasis placed on barley in the diet.

Generally, the owner or one of his sons worked the mule (Table 1); it was rare for someone outside the family to work the mule but this did occur where the mule was owned by a village elder, willing to lend out his own mule. The relationship the handler has with the mule they work is also affected by other factors, including the fact that adolescent boys today are away from home at school and this reduces the time spent developing a relationship with their family’s mule. They may then find themselves working the mule in school holidays and on weekends or when they have finished their schooling without having established a connection. Poor relations can be further aggravated where muleteering is not their preferred line of work and resentment is taken out on the mule (31). This is part of the break in inherited relational practice referred to earlier. The relationship that can arise when an owner owns, values, knows and appreciates his mule is not the same as that arising when a youth who has not invested his own funds and self in the mule finds himself having to work the mule.

Homecare was typically provided by the women of the family^32^ when feeding the livestock (cows and sheep) living with the mule. No mules in either village had free access to water at home. Water was either made available when the mule went out to work or brought to them by bucket. Grain is typically fed twice daily in a nose bag made from plastic flour bags and is never soaked. In Aremd, some grazing is available above the village and mules are taken up there in groups during the winter months when work in tourism ceases. Many mules are, however, fed at home with womenfolk heading out every morning to scythe, collect and carry back fodder for all their livestock. This task is repeated again in the evening. They do so, bent double under the load for they do not work with the mules. These cultural norms are reflected in the title of a recent book on hidden injustices in Morocco: “Dos de femme, dos de mulet” (73). Montanari in her work on the future of agriculture in the High Atlas highlights the extent to which women have to work long hours collecting fodder for their animals (17, pp., 53, 54, and, 57, 58) and the challenges of sourcing fodder during the winter months. These practices and challenges were common to both villages in this study and can lead to mules being sold before the winter months to avoid the cost associated with feeding them when they are not working. Alfalfa is often fed to milk-producing cattle but its production locally is very limited and it is not offered to mules, who are primarily offered chopped straw as fodder. The use of straw and, more generally, cereal crop by-products as the sole feed source for draught animals is a common practice in many rural communities in the Mediterranean and Balkan regions. These feeds are characterised by poor nutritional value, due to the high content of indigestible fibre and this can compromise work efficiency. In the long term, the feeding of imbalanced, cereal straw-based diets can lead to poor health and metabolic disorders, such as increased calcium removal from the bones by oxalic acid, which is particularly abundant in cereal by-products (74). In Aremd, straw is largely brought in by lorry and this is coordinated by the local muleteer association. This practice has arisen because of the large number of mules and because the terraces that would previously have been used to grow wheat and barley are now largely given over to apple and cherry production (19).

Work undertaken by mules in both villages consisted of transportation of building materials, firewood, manure and agricultural produce, other household items as well as work in tourism. Mules in TO were significantly more likely to be used for construction work than mules in AR (*χ*^2^ = 15.35, *p* = 0.0040). All animals in both villages were used for tourism purposes, but none of the mules in TO were used exclusively for tourism purposes whilst work in tourism was the most important form of work in AR, with some mules only used in this sector (Table 1). Two forms of tourism work are recognised. The first involves local work and caters for visitors wanting to undertake local excursions. The local muleteer association operate a system whereby each of the four villages in Imlil are rotated for work; this means mules can expect to work 1 day in four. The second involves multi-day trips into the high mountains. This work is coordinated by the amine for each of the four villages.

### Wounds and other injuries

Wounds were identified as a significant welfare concern in both villages (Table 2) and were, in all cases, associated with part of the tack – either the pack saddle, the tethering system or the bit and bridle. In Aremd, 74.4% of mules had evidence of fresh or old wounds; by contrast, 55.6% of mules in TO had fresh or old wounds.

**Table 2:** Tack practices and wounds in mules between Aremd and Tizi Oussem.

		AR	TO
Wounds present	Yes	29	2
	No	11	4
Bit type used	Traditional	19	15
	Modern	11	1
	Both	2	0
Pack fit	Tight	20	2
	Not tight	12	2

**Figure 8:**
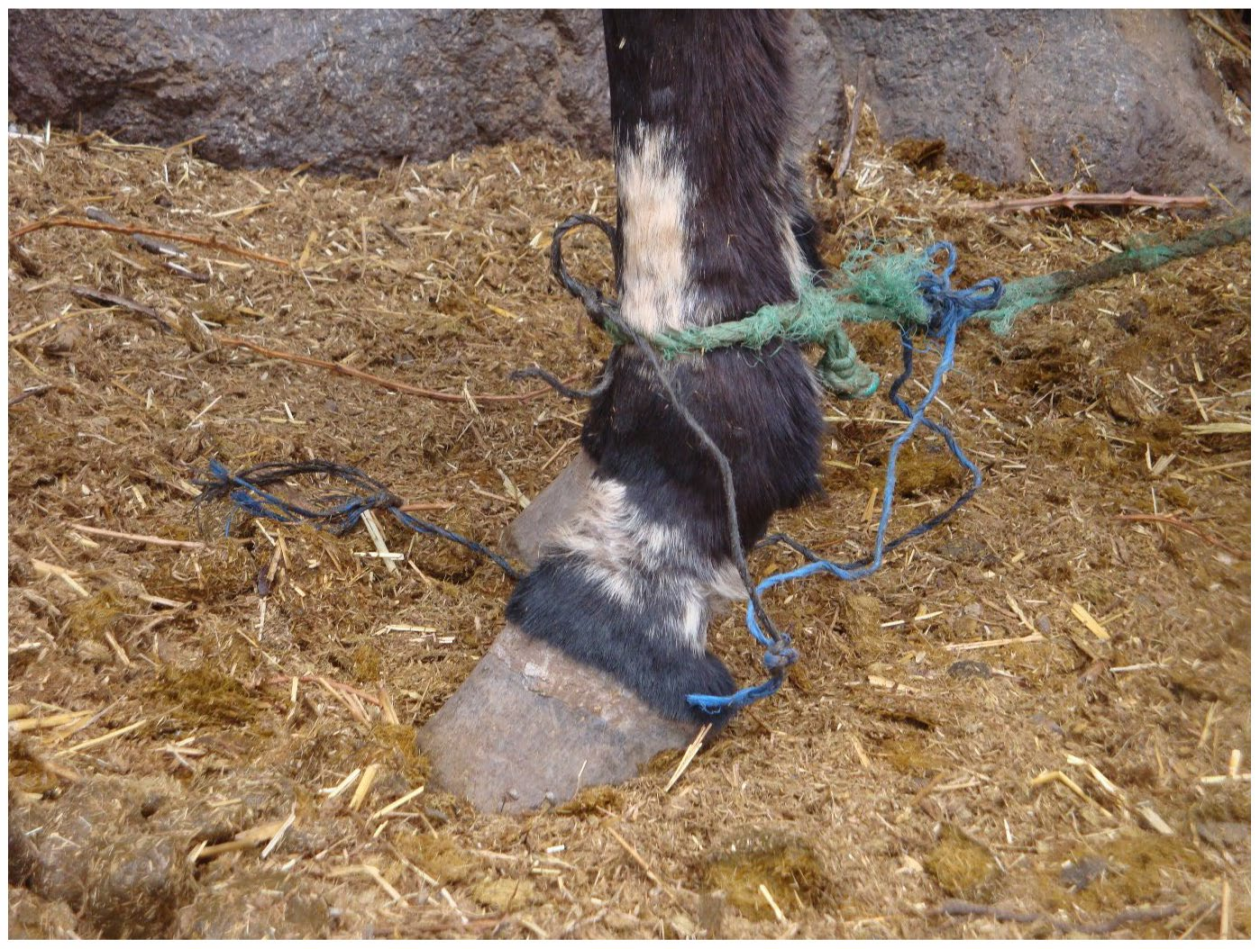
Mule tethered in Aremd in outdoor stabling area. Note the use of a single thickness of nylon rope and the loop of very thin nylon string on the ground that has also been used as a tether. Note also the extensive depigmentation of the hair over the pastern and cannon bone reflecting deep trauma to the dermis.

Wounds relating to the pack saddle were identified at the level of the withers, tuber ilium, elbow, back of the thigh, dorsal midline, under the tail and the girth band. Potential factors that might predispose a mule to packing injuries include poor body condition, poor conformation and poorly fitted and maintained pack saddles (11–13), together with the lack of a grooming practice. Wounds relating to tethering were identified primarily at the level of the pastern but also the cannon bone (Figure 8). Such injuries are common place across Morocco (11, 14, 73) and result from tethering and hobbling practices, which should be carefully distinguished and can easily be prevented (14, 75, 76).

**Figure 9:**
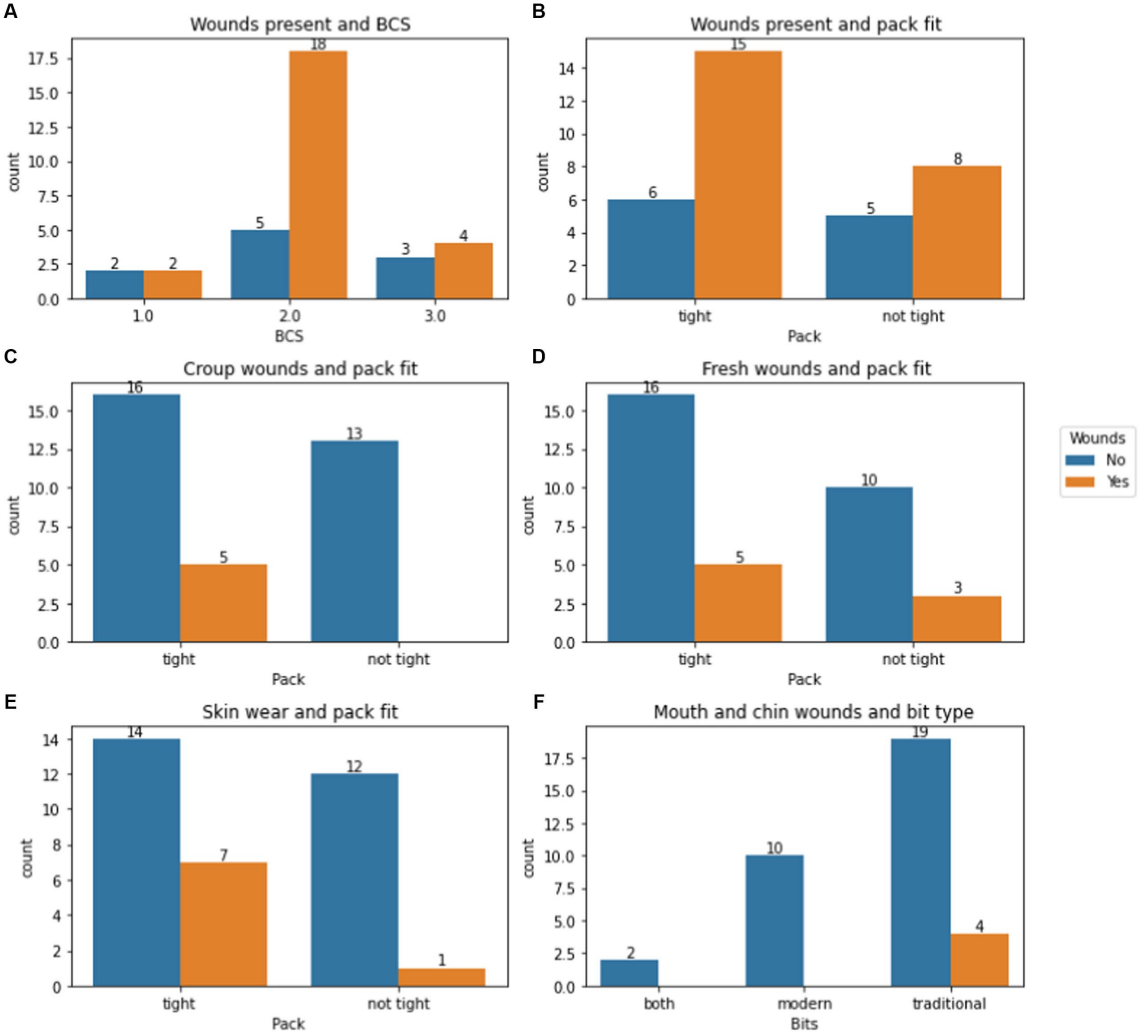
Bar charts illustrating number of mules with: **(A)** old or fresh wounds present, by BCS (*n* TO = 5; AR = 29); **(B)** old or fresh wounds present, by pack fit (*n* TO = 3; AR = 31); **(C)** croup wounds present, by pack fit (*n* TO = 3; AR = 31); **(D)** fresh wounds present, by pack fit (*n* TO = 3; AR = 31); **(E)** presence of wear to the skin, by pack fit (*n* TO = 3; AR = 31); and **(F)** mouth or chin wounds present, by bitting type (*n* TO = 3; AR = 31). The number of mules in each group is given above each bar.

**Figure 10:**
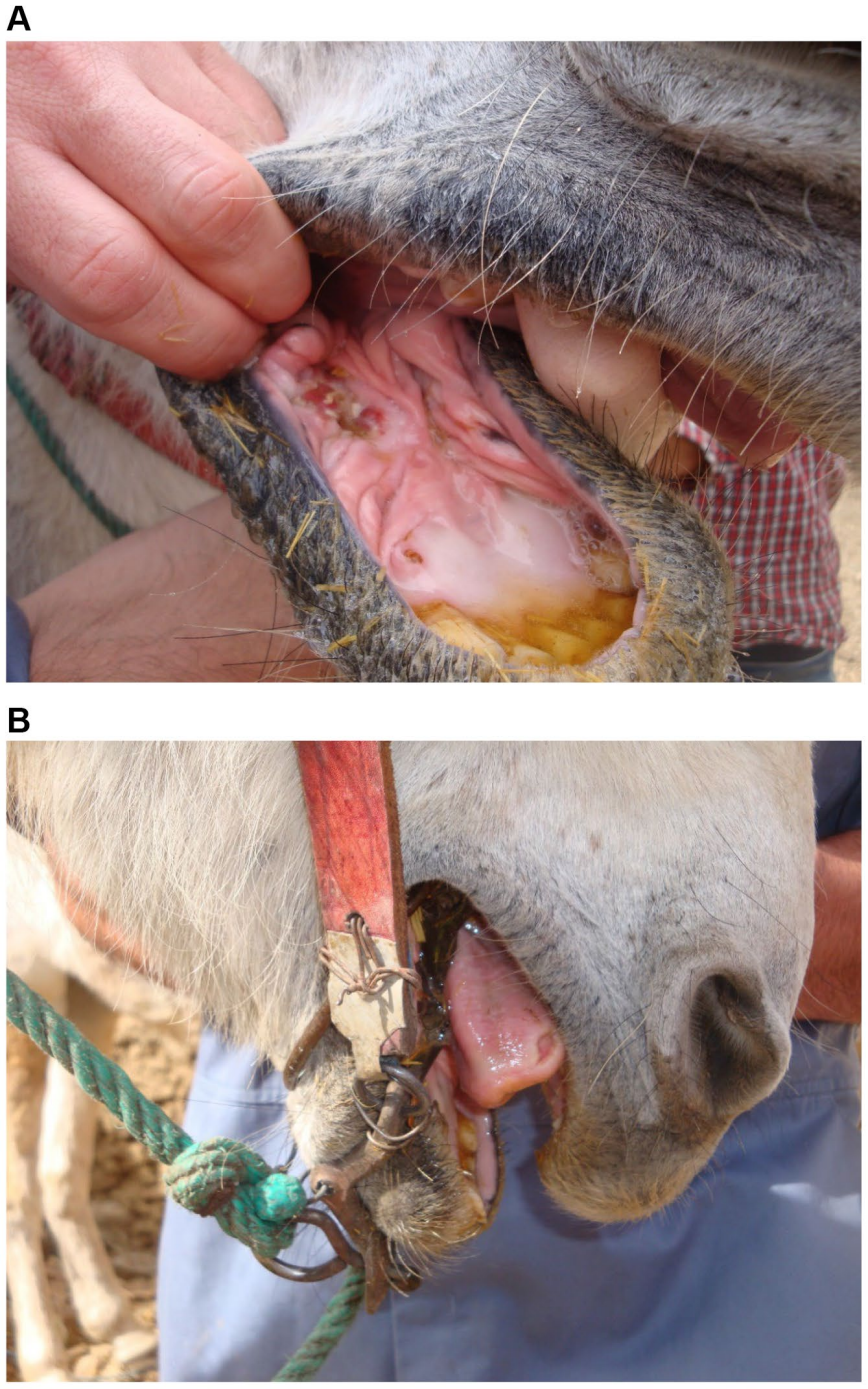
**(A)** and **(B)** The grey mule pictured here, from Tizi Oussem, shows injuries to both the bars of the mouth and tongue. The image below shows the use of metal wire to secure the leather bridle to the bit.

All mules and both donkeys in TO were worked in traditional bits, with one exception (Table 2). In this case, the owner had purchased a stainless steel bit from someone in Imlil. By contrast, in AR, 40.6% of mules used the modern bit supplied by SPANA at least part of the time. No mules were worked in head collars in either village. The reason for favouring the traditional bit was repeatedly given as the mule being difficult to work without the owner being able to control the mule through this device. Bitting injuries were more commonly seen and most common and severe where the mule was worked in a traditional bit (Figure 9). Injuries were identified to the bars of the mouth (Figure 10A), roof of the mouth, sublingual tissues and tongue (Figure 10B) as well as to the underside of the jaw. Injuries were due largely to the damage caused by a thin metallic bitting device being used to subdue, control and direct the mule with little or no awareness of the damage caused. Some injuries were the result of metal wire being used to repair the bridle and/or attach the bit to the bridle (Figure 10B).

### Hoof pathologies and hoof care

**Figure 11:**
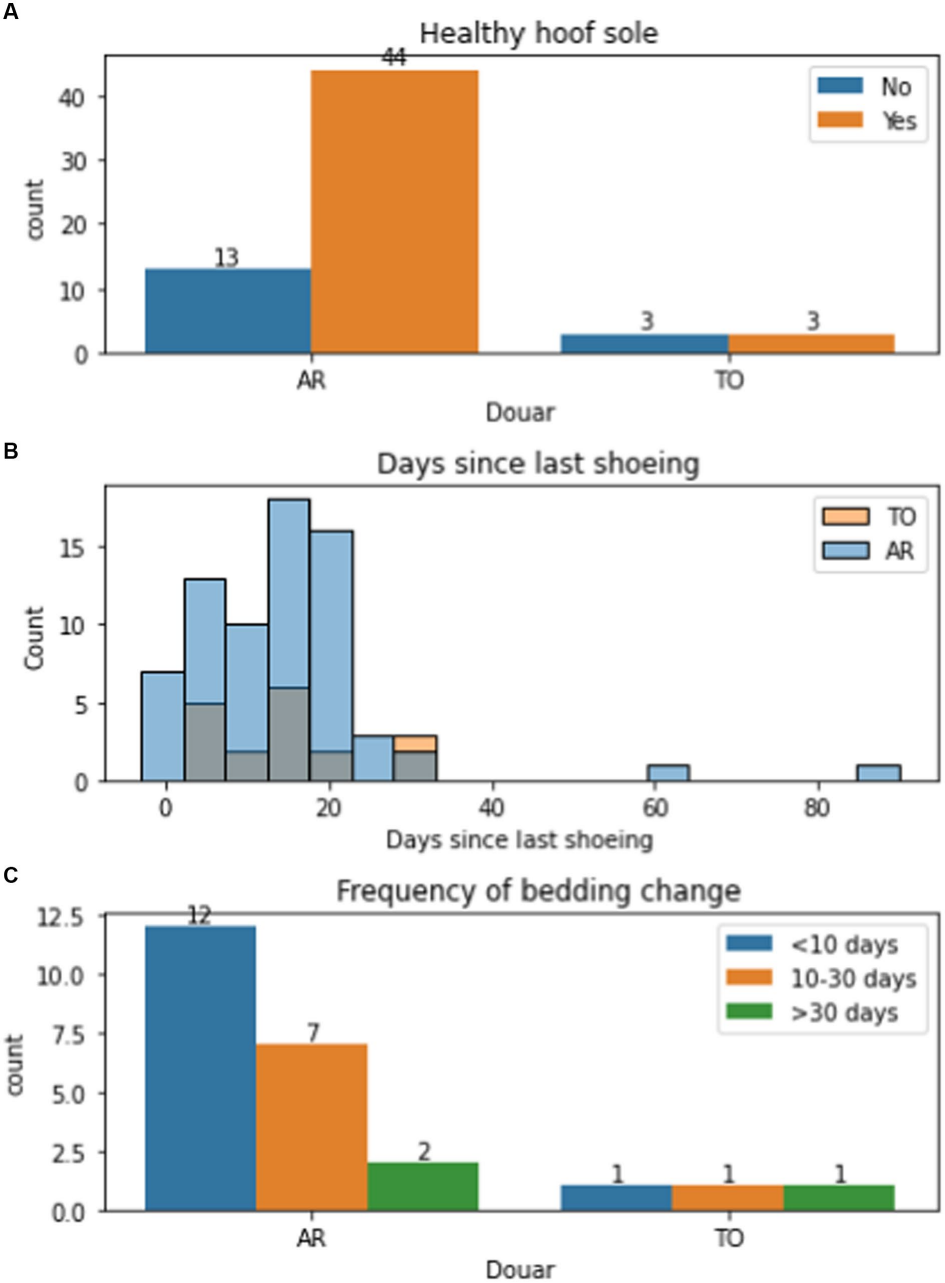
**(A)** Presence of healthy foot sole by Douar (village; Aremd (AR) and Tizi Oussem (TO)), with number per group given above the bar; **(B)** layered histogram of days since last shoeing in AR and TO; and **(C)** frequency of bedding change in AR and TO, with number per group given above the bar.

**Figure 12:**
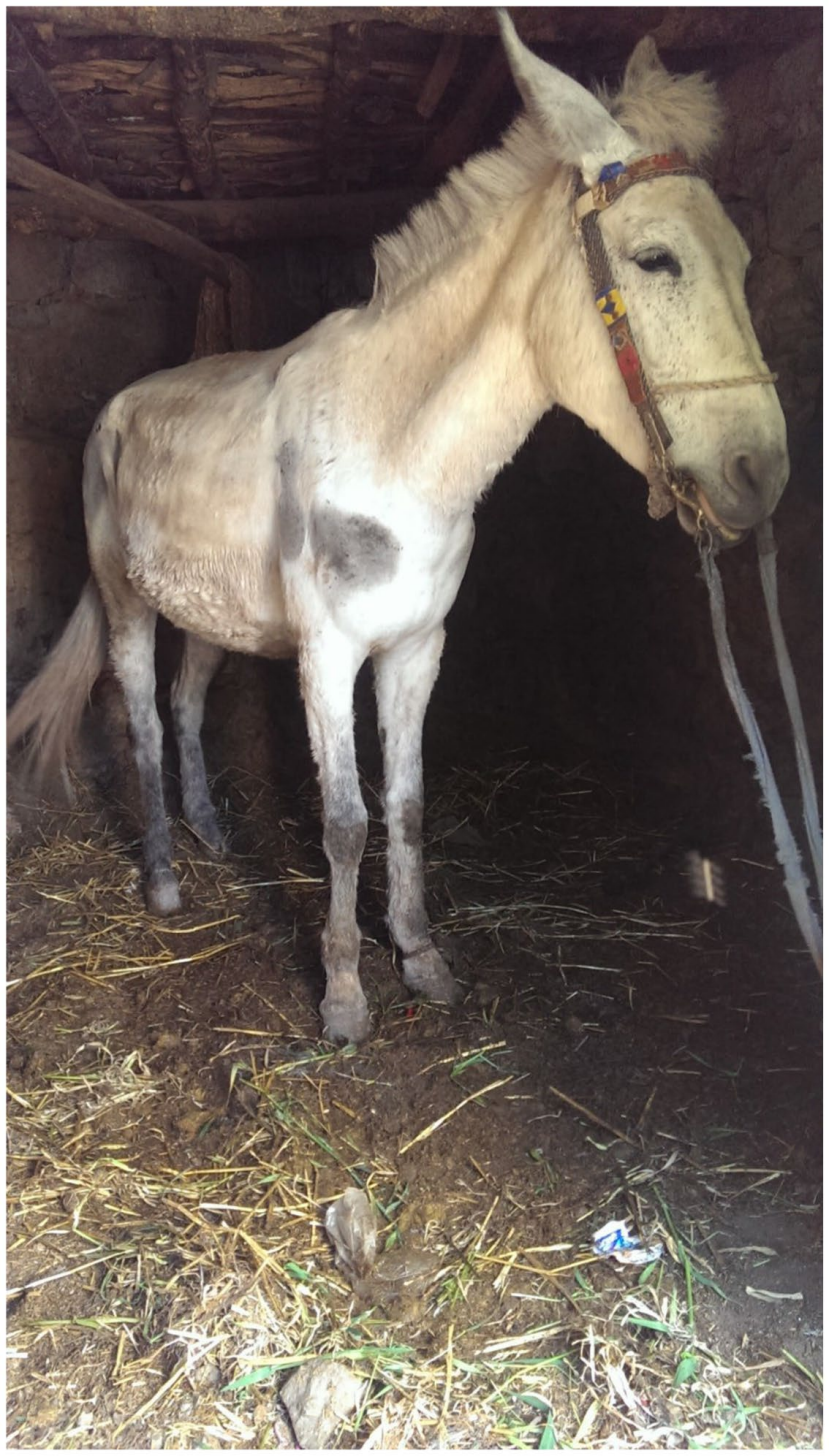
Mules are stabled overnight and through the winter in ground level stables. The manure is allowed to build up and is believed to keep the stable warm. Mules are typically tethered by the foot and not free to move around, often because owners find it easier to catch them this way. This reflects the lack of connection, mutual trust and respect between the handler and their mule.

**Figure 13:**
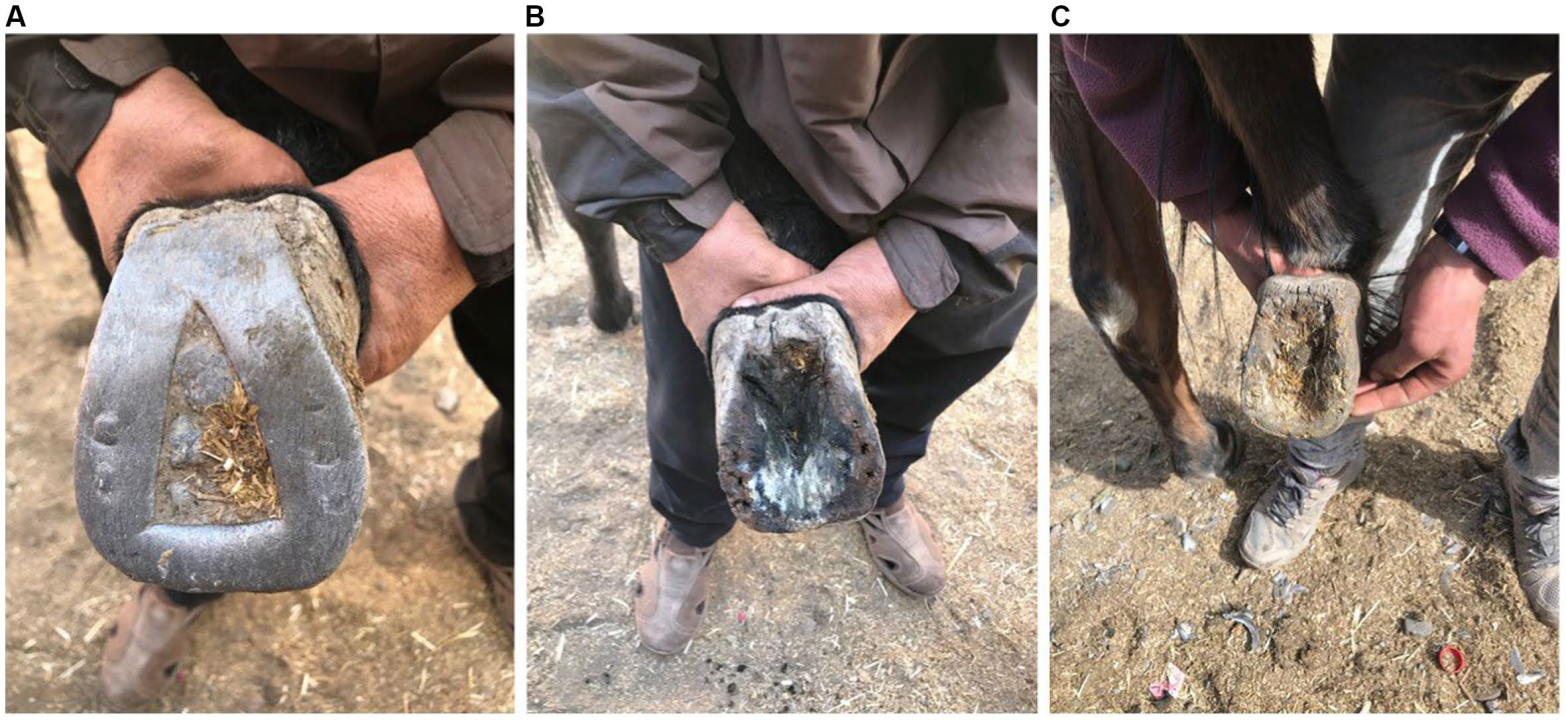
The traditional closed shoe is bent over the heel and does not allow healthy functioning of the frog **(A)**. It allows soiled bedding and other material to become trapped around the frog leading to maceration of the sole **(B)** and degeneration of the frog (**B,C**).

All mule owners used traditional farriery methods with closed shoes applied to the hoof by the local blacksmith. Shoeing was undertaken at a similar frequency in both Douars (mean time since last shoeing was 14.3 days in AR and 14.7 in TO) (Figure 11). This meant that hooves were not subject to wear when working on local terrain. The shoeing involved the use of a traditional cleaving tool that cut a wedge of horn from the foot in a sweeping action from heel to toe prior to applying new shoes, but did not involve any balancing of the foot (see Figure 11). As a result, many mules presented with a long toe, short heel conformation, with toe dumping a common practice. The closed nature of the shoe, traps material in the frog and makes hoof care difficult. There was no awareness of hoof hygiene – feet were not picked out and cleaned and mules were typically bedded in their own manure (Figure 12) because this was believed to keep the stable warm and because there is no litter (e.g., straw) available. During the winter months, mules are typically confined to their stables and their feet are therefore immersed in manure and urine. When manure was used as a bedding substrate, mules were significantly more likely to have an unhealthy hoof sole (*χ*^2^ = 14.29, *p* = 0.0064), with foot rot the most common finding (12 mules) leading to degeneration of the frog (Table 3, Figure 13). This is exacerbated by contracted heels and excessive trimming of the frog (77, p. 379). These factors mean that the hoof does not have opportunities to find its own natural healthy balance (78) and become resistant to abrasion and other insults. Instead, the sole has a tendency to become soft and vulnerable and owners have little confidence in the hoof’s natural defences, believing that the heel needs to be kept covered and protected.

**Table 3:** Healthy hoof sole and associated management factors in mules.

		Healthy hoof sole	Unhealthy hoof sole
Bedding state	Dry	15	5
	Wet	1	8
Manure used as bedding	Yes	5	12
	No	15	1
Frequency of bedding change	<10 days	9	3
	10–30 days	2	5
	>30 days	2	1
Frequency of mucking out	Daily or less	2	0
	2–10 days	6	0
	10–15 days	2	4
	>15 days	6	7

## Concluding discussion

This paper has traced the various ways that local communities on the Toubkal have come to interact with the outside world, acquire and start working mules. Initially, mules were only owned by a few individuals. This gradually changed as opportunities emerged to earn money that rendered mules affordable and serviceable. With the advent of mountain tourism, the mule population in Aremd has grown substantially and now most families have a mule who is utilised to generate income wherever opportunities arise to do so. These developments have not been accompanied by improvements in mule care and awareness of both mulemanship and the complex network of actors contributing to mule welfare issues is limited (10, 11, 13, 79).

Each of the welfare issues reported here – poor body condition, pack wounds, tethering injuries, bitting injuries, and foot pathology are the product of a constellation of relational (10) socio-cultural, historical, geographical, technical, material, educational, and economic factors (80). A systems approach to such problematics requires us to recognise the need to see beyond the presenting surface phenomena to the deeper elements of an iceberg model: the patterns of behaviour, the ways parts are related, structure the system and influence the patterns and the underlying mental models, as well as the values and beliefs that shape the system (80). We also need to recognise that the transmission of knowledge is both vertical between members of different generations, stereotypically between parents and their offspring but also horizontal between the members of the same generation (17, p. 58). This further requires us to recognise the complexity of such phenomena and adopt a collaborative and participatory approach (13, 14, 81) that moves beyond knowledge transfer (82) and into awareness-based systems change, recognising the need to address attitudes, mind sets, beliefs and mental models through cycles of facilitated reflection and action (10, 13, 31, 80, 81) as well as the more obvious practicalities that traditional veterinary health care interventions focus on. In the case of the system within and from which mule welfare arises, there needs to be recognition of the shared responsibilities of the stakeholders who, as a network, co-create mule welfare. The mining and mountain tourism industries have a long history of hiding human and animal welfare abuses underground and in other ways (6, 83–87). These include the abuses that lie hidden in the mouths (10) and under the pack saddles (13) of working equines as well as the abuses these animals are subject to when they are sold on to work in the cities. These issues are challenging, especially given the limited resources available to transform the system (see Figure 13).

A co-creative, collaborative approach seeks to realise the collective intelligence of the system (88, 89) and this means creating holding spaces for change to emerge (31) with those who are most open to delivering these emergent futures (77, 90) being supported to prototype new ways of relating and working with pack mules that transcend the disinterest, lack of compassion and fear-based absencing (10) that characterises current relations. Whilst swapping traditional bits out for modern stainless bits may be helpful (91) in addressing the worst of the injuries, it is important to recognise that these technical answers do not address underlying root causes. A systems approach (92–94) informed by detailed ethnographic understanding of relational working practices recognises that the relationships between many mules and their handlers are fear based and that training is required to facilitate a shift from domination to partnership (10, 95). As part of this it is helpful to recognise that head collars are more appropriate for ground work where the mule is unridden (10, 96). Owners can be taught basic shaping behaviours using positive reinforcement techniques that over time will allow mules to be handled easily and safely (31, 97). This takes times but is especially important as regular exposure to stress-free handling and to cleaning of the feet habituates mules to such interactions. Daily grooming and foot care also allow rubs, wounds and other health problems to be identified early.

Tourists themselves have a role to play by exercising their discretion in choosing service providers and by taking an active interest in the welfare of the mule who they travel with. Tourists are also increasingly reporting their concerns (90) in ways that demonstrate the importance of working equid welfare in tourism (7). Trekking agencies are able to write welfare specifications into their contracts with local agencies (13) and can develop and subscribe to industry codes of practice (96). Local authorities can introduce local regulations and NGOs such as SPANA can undertake technical inspections through their monthly visits to the area (91) and contribute to training initiatives, especially of local farriers, saddle makers and other artisans. Local muleteer associations have a crucial role to play in advocating for their members and professionalising the services they provide so that these revenues can be invested in better equipment, training and education. A key focus here, arguably needs to be on co-creating futures that are affordable and accessible whilst respecting mule welfare and the needs of the community. Such participatory and collaborative approaches are radical (97) and require significant capacity building and an investment in meeting and listening together. There is a clear role for research in this sector. Hossaini-Hilali reports that, between 1976–2011, only 28 scientific publications on equines were produced by students at the Institute Agronomique et Vétérinaire, from the 149 veterinary theses written during this time (98, p. 28). A shift in the kind of research undertaken is also needed if the inter-relationships between agriculture, mule welfare and tourism are to be unpacked. Allali, in his call for sustainable tourism, highlights the opportunity provided by seeing the impact of tourism on the local environment that arises when people work both in agriculture and tourism (99, p. 69). The reduction in the amount of cereal production arising from decisions to plant trees (19), with these occupying 80% of the agricultural area surveyed (100, p. 69) similarly shows how decisions in one sphere have impacts elsewhere – specifically on the food available to livestock. A study, similar to that reported recently from Kashmir (101) is thus needed to determine the volume and nutritional quality of the grain and fodder currently eaten by local mules and how the system can better provide for the nutritional needs of working pack mules. This could include payments in kind that go directly to the mule and that subvert exploitative practices.

In conclusion, there is a need for more collaborative partnerships across the mountain tourism system to allow communities and their mules to break free from the constraints of the limited means and rationalities, the narrow valleys, mind-sets and relational practices that have historically limited their ability to realise more optimal welfare.

## Data availability statement

The datasets presented in this article are not readily available because the animal data collected has been provided by SPANA Maroc. Requests to access the datasets should be directed to GC glen.cousquer@ed.ac.uk.

## Ethics statement

The ethnographic part of this study is reported retrospectively here. The original study was approved by the Ethical Committee of the University of Edinburgh’s School of Geosciences. The animal studies were approved by R(D)SVS Veterinary Ethics Research Committee. The studies were conducted in accordance with the local legislation and institutional requirements. Written informed consent was not obtained from the owners for the participation of their animals in this study because oral consent was deemed more appropriate given the cultural contexts in which the work was undertaken. Written informed consent was not obtained from the individual(s) for the publication of any potentially identifiable images or data included in this article because written consent is not deemed culturally appropriate and oral consent was used instead.

## Author contributions

GC: Conceptualization, Data curation, Formal analysis, Funding acquisition, Investigation, Methodology, Project administration, Resources, Supervision, Writing – original draft, Writing – review & editing. HA: Resources, Supervision, Writing – review & editing. VL-M: Formal analysis, Software, Writing – review & editing.

## Footnotes

^1^From references in secondary sources, Google Scholar and online archives of turn of the Century texts.

^2^Docteur de Médecine Vétérinaire.

^3^Calculated from the girth and distance between the elbow and tail base.

^4^See Rachik (2012) (34) for an exquisite review of the different ways in which travellers have come to know Morocco.

^5^Delaye was put in charge of the aerial photography section of the ‘*Service géographique du Maroc*’ in 1926. His pioneering work, first in the Rif (1925–26) and subsequently in the regions of Ouarzazate and the Drâa valley (1932) and then the Sagho (1935), allowed the occupying forces to pacify the mountain tribes [46;47]. In 1937, he produced a map of the Toubkal massif at a scale of 1:20,000 (51–53).

^6^The Tizi Tar’ret. as he spells it had according to Penet (45, p.10) already been crossed first by Von Fritsch, then Gentil but had seen Brives renounce his attempt due to the poor reception (“mauvaise disposition”) of the people of Aremd.

^7^Translated from French by the author (GC).

^8^At an exchange rate of 13 dirhams to £1.

^9^The completion of the piste is reported in the news section of Montagnes Marocaines, the journal of the Casablanca section of the Club Alpin Français. Until that point, the amine who was contacted to organise mules for a trek in the valley was located in Asni.

^10^Tidli is the name given to the area above Aremd. According to Hajj Morris (personal communication 31/08/14), the name of the mine above Aremd was ‘Couchar’, whilst the bigger one above Tamatert was called ‘Talat n Taghsen’.

^11^Equivalent to £1.30 (at an exchange rate of 13 dirhams to £1).

^12^Field Notes 18, page 8 (30^th^ August, 2014).

^13^Field Notes 19, page 19 (2^nd^ September, 2014).

^14^For further information on the introduction of fruit trees to the valley see Funnell and Parish (19).

^15^Mules can often follow familiar paths in the dark of night, so well do they attend to their passage. The history of mining is also etched into the memories of the old men of the valley, many of whom were reduced to seeking work there when they were not needed in the fields or to tend their livestock.

^16^Field Notes 19, page 24 (2^nd^ September, 2014).

^17^Field Notes 18, page 8 (30^th^ August, 2014) and Field Notes 19, page 30 (2^nd^ September, 2014).

^18^Field Notes 19, page 24 (2^nd^ September, 2014).

^19^Recent changes to Moroccan legislation mean that this covers not just his wife and children but also his parents.

^20^Field Notes 18, page 4 (29^th^ August, 2014). Interview with Fabrice Cuzin, consultant biologist, ex-manager of the CAF refuges and instructor at the CFAMM. Translation: «La transmission est. cassée … les anciens ne représentent plus de référence pour les jeunes! … Il n’y a pas d’apprentissage!»

^21^Details for B&Bs and Inns checked on the 6^th^ September, 2017. This excludes hotels.

^22^In the case of walnut harvesters, this is recognised as dangerous work, with workers having to scale the trees and move through the canopy to knock walnuts to the ground. As a result, it attracts a daily rate of 300 Mad per day with the walnuts selling for 200–50 MAD per thousand. By comparison, a daily rate for apple harvesters is 100 MAD.

^23^Within this, it is important to distinguish between three forms of tourism: that involving foreign trekkers, that involving locals escaping the city and that involving locals undertaking pilgrimages (67, p. 6).

^24^Interview with Lahcen, April, 2015.

^25^These rates are from 2014–2017. With the creation of a new muleteer association for the valley, covering the villages of Aremd, Mzik, Aït Souka and Imlil itself, a revised fixed set of rates have been introduced.

^26^Field Notes 4, page 5 (25^th^ March, 2014). Translation: “I did not find her in a box of Laughing Cow cheese.”

^27^Interview with Lahcen, April, 2015.

^28^Field Notes 43, page 23 (21^st^ October, 2015).

^29^Field Notes 35, page 11 (3^rd^ June, 2015). Translation: “You know the area, you know the people. You will find someone who is poor … who buys a mule for 3,000 dirhams … a bad mule … But what does he do? He needs a mule to find work … to keep his family. He is only thinking of earning a little money and he goes without thinking about the mule. You know the mules who have died in the mountains. These are the mules of poor men, the men who have bought their mules for 3,000 dirhams, 4,000 dirhams.” A good mule can, by comparison, be expected to cost in excess of 15,000 dirhams.

^30^Translation: “Many have concluded that this people had no knowledge of equestrian principles, refusing them even all love of the horse. This results from a failure to recognise that to save their families, to preserve their property and often in obedience to the laws of Jihad, these same Arabs … were forced to use their horses according to the needs they were subject to and prevailing circumstances but they knew perfectly well that it was preferable not to act thus.”

^31^According to the village associations.

^32^Who often do not pursue their studies after their junior school years because going to the college requires them to travel to Asni, the nearest town.
